# Clinical outcomes of free gingival graft vs. palatal pedicle graft in peri-implant soft tissue phenotype modification: A randomized controlled trial comparing patient reports

**DOI:** 10.34172/japid.2024.016

**Published:** 2024-08-12

**Authors:** Hossein Khoshkhou, Siamak Yaghobee, Mohammadjavad Kharrazi Fard, Mahsa Etemadi, Seyed Hossein Mohseni Salehi Monfared

**Affiliations:** ^1^Department of Periodontics, School of Dentistry, Tehran University of Medical Sciences, Tehran, Iran; ^2^Department of Periodontics, Dental Implant Research Center, Tehran University of Medical Sciences, Tehran, Iran; ^3^Dental Research Center, Tehran University of Medical Sciences, Tehran, Iran; ^4^Department of Periodontics, School of Dentistry, Tehran University of Medical Sciences, Tehran, Iran

**Keywords:** Dental implants, Free gingival graft, Keratinized tissue width, Palatal pedicle graft, Surface shrinkage

## Abstract

**Background.:**

The importance of peri-implant soft tissues in maintaining tissue health and aesthetics has been recognized. A thickness of at least 2 mm is considered a protective factor against peri-implantitis. This study assessed clinical outcomes and complications at implant sites following soft tissue augmentation with either palatal free gingival graft (FGG) or palatal pedicle graft (PPG).

**Methods.:**

In this randomized controlled clinical trial, 42 patients with inadequate keratinized tissue width (KTW) were randomly assigned to two intervention groups: Group 1 received FGGs, while group 2 underwent PPGs. The KTW, vestibular depth, and surface shrinkage were recorded preoperatively and one and three months after the operation. Patient-reported outcome measurements were recorded at a two-week follow-up.

**Results.:**

Thirty-five patients completed the study (FGG group, n=17; PPG group, n=18). Group 2 demonstrated a higher increase in KTW and vestibular depth at 1 and 3 months (*P*<0.05). The surface shrinkage differences were not statistically significant between the study groups at baseline and three-month follow-up (*P*>0.05). The number of analgesics in each group was not significantly different two weeks after the operation; however, the Numeric Pain Rating Scale (NPRS) showed significantly higher pain scores on days 3 to 8 in group 1 patients.

**Conclusion.:**

The use of PPG in soft tissue augmentation demonstrated more KTW formation and less postoperative morbidity. There was no difference between the methods used to compare surface shrinkage.

## Introduction

 Dental implants have revolutionized dentistry by providing reliable treatment options for replacing missing teeth. Although dental prostheses are commonly used, patients often remain dissatisfied with the aesthetic and functional reconstruction of their oral cavity. As a result, many patients opt for implant treatments instead.^1^ The long-term survival of dental implants depends on peri-implant hard and soft tissue maintenance. As a result, it is of utmost importance to maintain peri-implant tissue health following implant placement by implementing a comprehensive check-up protocol and supportive therapy.^2^

 Over the past few decades, the importance of peri-implant soft tissues in maintaining tissue health and aesthetics has been recognized. A keratinized mucosal thickness of at least 2 mm is considered a protective factor against peri-implantitis, and its lack has been introduced as a risk indicator of peri-implant mucositis severity.^3^ An insufficient keratinized mucus around the implant is associated with greater plaque accumulation, soft tissue inflammation, and gingival recession.^4^ Moreover, evidence has suggested that reduced keratinized mucosal width ( < 2 mm) is associated with patient discomfort, improper plaque control, the possibility of marginal bone loss, and bleeding on probing.^5^

 Soft tissue augmentation has recently been proposed as a viable strategy to improve the long-term success and clinical and esthetic outcomes of dental implant restorations.^6^ A systematic review showed that soft tissue modification with a free gingival graft (FGG) is the most effective technique in increasing the width of keratinized mucosa.^7^ FGGs are successful and predictable; however, they have some disadvantages: two surgical sites are involved, with the corresponding morbidity in both areas. It provides a limited amount of tissue volume.^8,9^ Additionally, color and texture discrepancies with the surrounding mucosa often compromise esthetic outcomes.^10^

 The palatally advanced flap is a useful, fast, easy-to-perform surgical technique for immediate implant placement in the maxilla. This approach ensures sufficient tissue bulk and mobility to the flap. This enables complete, precise, and highly predictable coverage of the extraction area, even for large defects requiring regenerative therapy and those needing multiple implants. The palatal tissue provides an abundant blood supply. Moreover, keratinized tissue is bridged over the implant site without disrupting normal anatomical relationships in the buccal area.^11^

 This study aimed to compare the clinical and postoperative outcomes of FGG and palatal pedicle graft (PPG) technique following peri-implant soft tissue augmentation.

## Methods

 This randomized, parallel-group clinical trial was conducted on 42 patients with insufficient keratinized tissue width (KTW) around the maxillary implant, referred to the Department of Periodontics, Faculty of Dentistry, Tehran University of Medical Sciences. This study was reviewed and approved by the Ethics Committee of Tehran University of Medical Sciences (IR.TUMS.DENTISTRY.REC.1401.079). The protocol of this trial was also registered in the Iranian registry of clinical trials with the code IRCT20221226056930N1. All the included patients agreed to participate in this investigation, signing an informed consent considering the 1975 Declaration of Helsinki, revised in 2013.

###  Participants

 According to the results of Goldstein and colleagues’^11^ study and two-sample t-test analysis considering α = 0.05 and β = 0.2, the average standard deviation of the keratinized mucosa width was 0.98 to discover a significant difference of 1 mm. The minimum required sample volume in each group was n = 17. Notably, the volume necessary for other dependent variables was less than this amount.­ The main eligibility criteria were as follows: (a) no less than 18 years of age, (b) generally and periodontally healthy patients with no medical contraindication for tissue augmentation surgery, (c) keratinized tissue of less than 2 mm apicocoronal width around implants, (d) a minimum of 2 mm of keratinized tissue at the palate, (e) 1-3 non-loaded bone-level cemented implants at maxilla, (f) implants with adequate primary stability (torque ≥ 35 Ncm). Patients with the following criteria were excluded from the study: (a) a history of radiotherapy, active periodontitis, or other signs of inflammation, infection, conditions, or drugs that adversely affect the periodontal status and comprise wound healing, (b) pregnancy or lactating women, (c) smokers ( ≥ 10 cigarettes per day), (d) alcoholism and drug addiction, (e) poor oral hygiene, (f) history of previous tissue augmentation at the region.

###  Randomization and blinding

 A randomization list was used to assign participants to treatment groups (FGG or PPG). The random allocation table was generated by balanced block randomization. The type of intervention was recorded in sealed envelopes. Surgeons received sealed envelopes numbered in order by the practitioner just before surgery. Blinding the patients and surgeons was impossible as both could discern the outcomes of the surgery; however, they were not aware of the allocation process. A practitioner unaware of the intervention conducted the clinical examinations and calculated the tissue shrinkage using ImageJ software (https://imagej.nih.gov/ij/download.html). A blinded statistician, unaware of the intervention and allocation processes, analyzed the data.

###  Outcome measures

 Primary clinical outcomes were dimensional changes in the apicocoronal KTW, vestibular depth, and vertical tissue shrinkage. Postoperative morbidity based on patients’ reports was also evaluated based on the number of painkillers taken by the patient during the 14 days after the surgery and the recipient and donor site morbidity using the Numeric Pain Rating Scale (NPRS).

###  Surgical procedures

**Figure 1:**
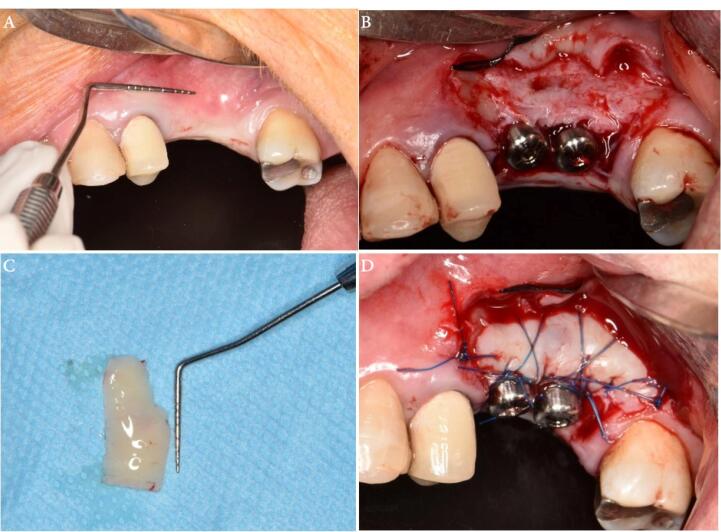
The surgical procedure. (A) Insufficient keratinized tissue at the implant site using the roll technique. (B) Recipient site preparation and healing abutment placement. (C) Free gingival graft size measurement. (D) Stabilization of free gingival graft with periosteal and simple loop sutures

**Figure 2:**
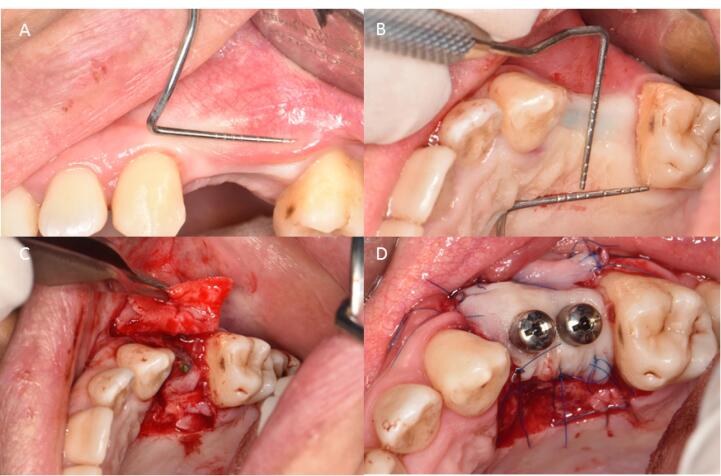
The surgical procedure. (A) Insufficient keratinized tissue at the implant site using the roll technique. (B) Pedicle flap size measurements. (C) Designing and reflecting the pedicle flap. (D) Stabilization of the flap with periosteal and simple loop sutures

 One week before surgery, all the participants received the necessary initial therapy, which involved oral hygiene instructions and scaling and root planing procedures to reduce periodontal pathogens to a minimum level. The patients were given one gram of amoxicillin one hour before surgery as antibiotic prophylaxis. Before the procedure, the patients were asked to rinse for 1 minute with 0.2% chlorhexidine mouthwash (Perio-Aid, Dentaid). A local anesthetic agent (2% Lidocaine, 1.8 mL with 1:100 000 epinephrine) (DaruPakhsh Pharmaceutical Mfg. Co., Tehran, Iran) was used for local infiltration of the edentulous area tissues. After sufficient anesthesia, the incision was made by a #15 scalpel. Initially, a horizontal incision was made at the mucogingival junction or 1 mm above it. This included the marginal gingiva/mucosa of the recipient site and was extended at least 3 mm in both the mesial and distal directions. Two vertical releasing incisions were made from the borders of this incision towards the alveolar mucosa. A split-thickness flap was carefully dissected to ensure adequate vascularization for the upcoming graft. The FGG (1.5 mm in thickness, 7 mm in width) was harvested from the palatal area and fixed to the recipient area by 5-0 nylon sutures by single interrupted and periosteal sutures (Figure 1). In the PPG group, a partial-thickness incision was made in the palatal region, depending on the size of the incision area. The partially elevated palatal graft was buccally fixed to the recipient area with a 5-0 nylon suture and a single interrupted suture (Figure 2).

###  Follow-up

 After the surgical procedure, all participants were prescribed analgesics (Gelofen, 400 mg, as long as required, at least every four hours) and antibiotics (Amoxicillin, 500 mg, three times daily for seven days) or (Clindamycin, 300 mg, four times daily for seven days) in cases of penicillin allergy. Oral hygiene instructions were given, and the patients were advised to start rinsing with physiological serum (normal saline, 0.9%) twice daily for two weeks, 24 hours after surgery. The patients were asked not to brush their teeth, apply pressure, or cause trauma to the surgical site. Two weeks after surgery, the sutures were removed. The patients were referred to prosthetic rehabilitation two months after surgery once the peri-implant tissues had completely healed.

###  Postoperative examinations

####  Patient-reported outcome measurements

 The level of pain and morbidity was evaluated in recipient and donor sites. Immediately after the surgery, a questionnaire was provided for the patients, and they were asked to score their pain from 0 (no pain) to 100 (unbearable pain) based on the NAS index. Also, the patients were asked to report the daily number of painkillers they consumed during 14 days after the surgery.

####  Clinical measurements

**Figure 3:**
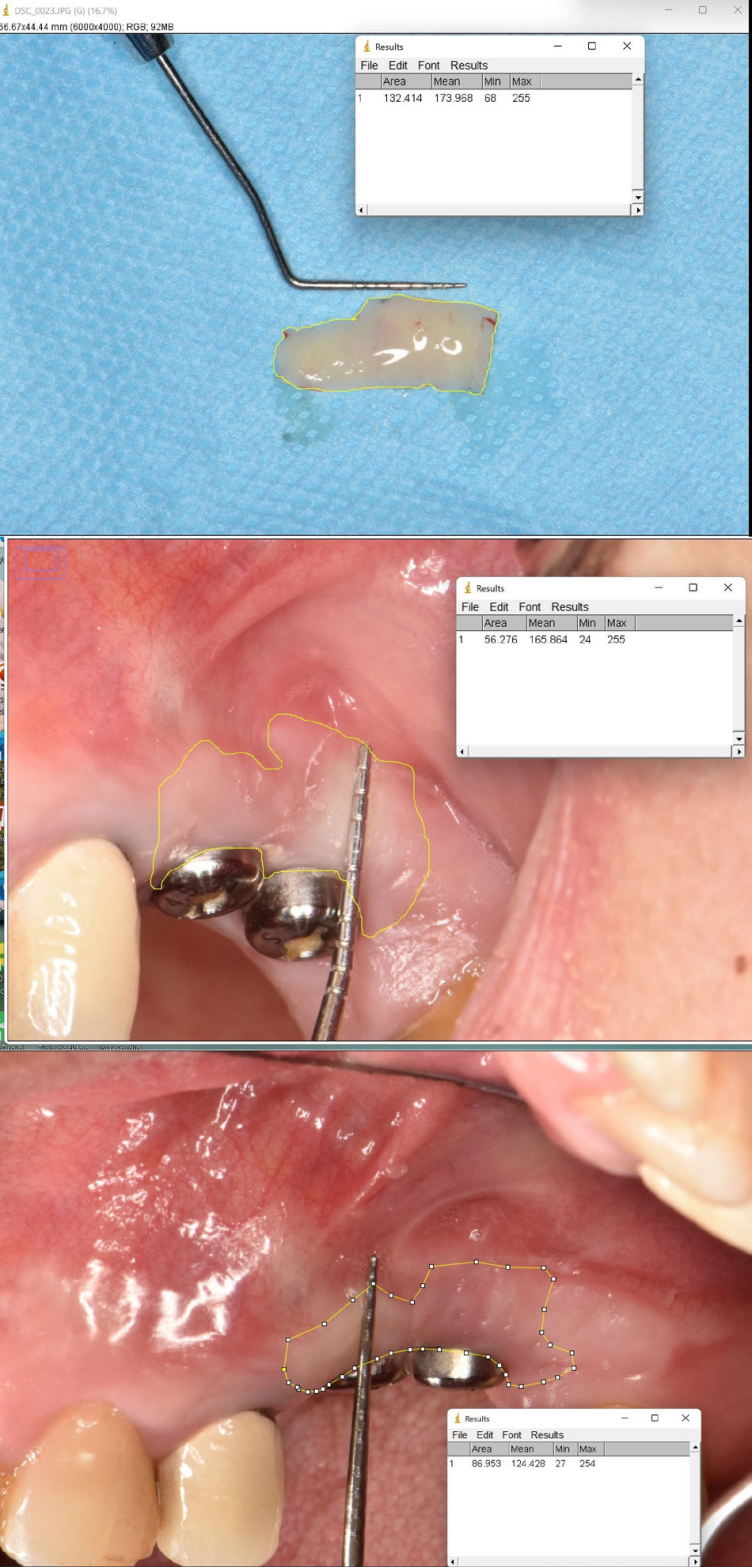
Tissue shrinkage calculation using the ImageJ software

 A single experienced clinician performed all the examinations. To evaluate the graft tissue shrinkage, the surface area of transplanted tissue was recorded at baseline and one and three months after surgery using ImageJ software (https://imagej.nih.gov/ij/) (Figure 3). The changes were reported in mm^2^. Similar to previous studies, the gingival margin in the mid-buccal region of the implant was considered the reference point for measuring the KTW.^12-14^ The apicocoronal width of keratinized tissue (mm) at baseline and 1- and 3-month intervals post-surgery was measured with a Michigan O probe by the roll test (UNC15). The depth of the vestibule (mm) was recorded by the Michigan O probe (UNC15) from the mid-buccal area of the implant to the functional depth of the vestibule at baseline and 1- and 3-month intervals after surgery.

###  Statistical analysis

 Statistical analysis was performed using SPSS 26 (SPSS Inc., Chicago, IL, USA). The Shapiro-Wilk test was used to determine the normality of data distribution (α = 0.05). The homogeneity of variance was confirmed by Levene’s test (*P* > 0.05). Quantitative variables with normal distribution were summarized as means and standard deviations, and the ones without normal distribution were reported as the interquartile range (IQR). In the case of parametric distribution, the t-test was used to detect differences between the groups. Mann-Whitney test was used to compare quantitative data with non-parametric distribution. A *P* value less than 0.05 was considered statistically significant.

## Results

**Figure 4:**
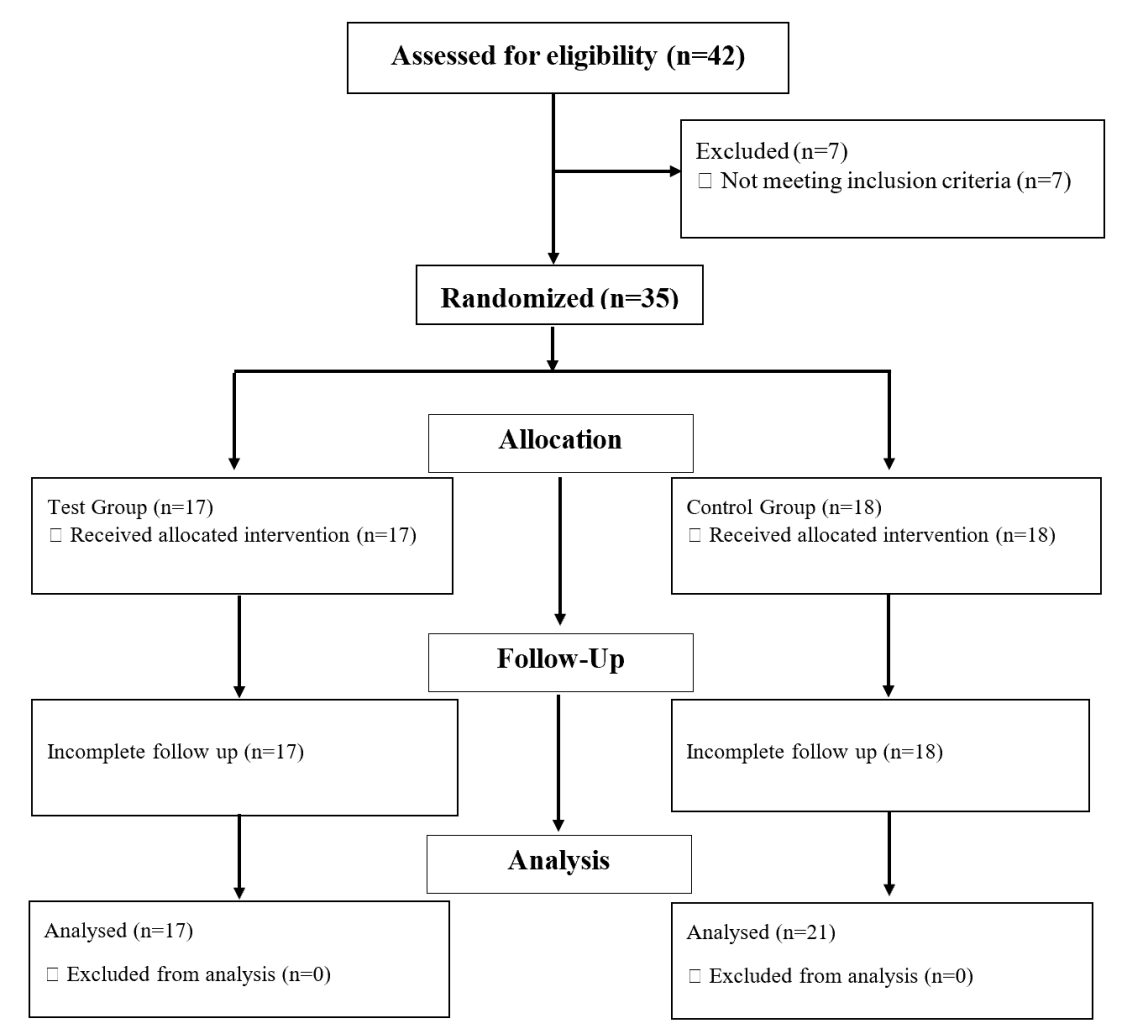
CONSORT flow chart of study participants

 Among 42 patients with maxillary implants, who referred to the Department of Periodontics, 35 patients (FGG = 17, PPG = 18) were included in the study based on the inclusion and exclusion criteria. No dropouts were registered during the three-month follow-up (Figure 4). The mean age of the subjects was 50.06 years (5 males and 12 females) in the FGG group and 52.11 years (5 males and 13 females) in the PPG group. The majority of participants were 28‒37 years of age. Table 1 shows the participants’ demographic characteristics.

**Table 1:** Descriptive findings of age and sex of the subjects

			**Gender**		**Total**
			**Female**	**Male**	
Group	FGG	Count	13	5	18
		% Within group	72.2%	27.8%	100.0%
	PPG	Count	12	5	17
		% Within group	70.6%	29.4%	100.0%
Total		Count	25	10	35
		% Within group	71.4%	28.6%	100.0%

FGG: free gingival graft, PPG: palatal pedicle graft.

###  Postoperative examinations

####  Patient-reported Outcome Measurements

 The average level of pain and discomfort was the highest on the first day of surgery, decreasing during the next few days. On days 3 to 8, the PPG group reported significantly less pain than the FGG group (*P* > 0.05), with no significant difference between the groups on other days (Table 2).

**Table 2:** Postoperative pain levels

**Day**	**Study group**	**N**	**Mean**	**Standard deviation**	**Standard error**	**95% Confidence Interval for Mean**	
						**Lower bound**	**Upper bound**
0	FGG	51	6.47	2.221	0.311	5.85	7.10
	PPG	66	5.70	3.296	0.406	4.89	6.51
	Total	117	6.03	2.892	0.267	5.50	6.56
1	FGG	51	5.76	2.103	0.295	5.17	6.36
	PPG	66	5.44	2.450	0.302	4.84	6.04
	Total	117	5.58	2.302	0.213	5.16	6.00
2	FGG	51	5.90	2.052	0.287	5.32	6.48
	PPG	66	5.05	2.508	0.309	4.43	5.66
	Total	117	5.42	2.350	0.217	4.99	5.85
3	FGG	51	5.57	2.156	0.302	4.96	6.18
	PPG	66	4.79	2.202	0.271	4.25	5.33
	Total	117	5.13	2.207	0.204	4.72	5.53
4	FGG	51	5.65	2.464	0.345	4.95	6.34
	PPG	66	5.59	2.511	0.309	4.97	6.21
	Total	117	5.62	2.480	0.229	5.16	6.07
5	FGG	51	5.57	2.532	0.355	4.86	6.28
	PPG	66	4.76	2.643	0.325	4.11	5.41
	Total	117	5.11	2.616	0.242	4.63	5.59
6	FGG	51	5.45	2.686	0.376	4.70	6.21
	PPG	66	3.74	2.574	0.317	3.11	4.38
	Total	117	4.49	2.747	0.254	3.98	4.99
7	FGG	51	4.61	2.442	0.342	3.92	5.29
	PPG	66	4.14	2.924	0.360	3.42	4.86
	Total	117	4.34	2.723	0.252	3.84	4.84
8	FGG	51	3.25	2.629	0.368	2.52	3.99
	PPG	66	3.05	2.330	0.287	2.47	3.62
	Total	117	3.14	2.456	0.227	2.69	3.59
9	FGG	51	3.22	2.809	0.393	2.43	4.01
	PPG	66	2.83	2.826	0.348	2.14	3.53
	Total	117	3.00	2.813	0.260	2.48	3.52
10	FGG	51	2.90	2.809	0.393	2.11	3.69
	PPG	66	2.32	2.322	0.286	1.75	2.89
	Total	117	2.57	2.551	0.236	2.11	3.04
11	FGG	51	2.06	2.509	0.351	1.35	2.76
	PPG	66	2.23	2.365	0.291	1.65	2.81
	Total	117	2.15	2.420	0.224	1.71	2.60
12	FGG	51	1.53	2.318	0.325	0.88	2.18
	PPG	66	1.48	2.032	0.250	0.99	1.98
	Total	117	1.50	2.152	0.199	1.11	1.90
13	FGG	51	1.29	1.781	0.249	0.79	1.80
	PPG	66	1.30	1.913	0.236	0.83	1.77
	Total	117	1.30	1.849	0.171	0.96	1.64

FGG: free gingival graft, PPG: palatal pedicle graft.

 During the first week following surgery, the greatest number of analgesics were consumed, decreasing from the first to the seventh day. There was no significant difference between the study groups except on day seven, on which more analgesic intake was reported in the FGG group (*P* = 0.001) (Table 3).

**Table 3:** Number of analgesics used after surgery

**Day**	**Study group**	**N**	**Mean**	**Standard deviation**	**Standard error**	**95% Confidence interval for mean**	
						**Lower bound**	**Upper bound**
0	FGG	51	2.961	0.8593	0.1203	2.719	3.202
	PPG	66	2.636	1.7154	0.2112	2.215	3.058
	Total	117	2.778	1.4118	0.1305	2.519	3.036
1	FGG	51	2.76	1.069	0.150	2.46	3.07
	PPG	66	2.53	1.571	0.193	2.14	2.92
	Total	117	2.63	1.375	0.127	2.38	2.88
2	FGG	51	2.78	1.189	0.166	2.45	3.12
	PPG	66	2.12	1.420	0.175	1.77	2.47
	Total	117	2.41	1.359	0.126	2.16	2.66
3	FGG	51	2.73	1.282	0.179	2.36	3.09
	PPG	66	1.89	1.337	0.165	1.57	2.22
	Total	117	2.26	1.372	0.127	2.01	2.51
4	FGG	51	2.61	1.613	0.226	2.15	3.06
	PPG	66	2.06	1.214	0.149	1.76	2.36
	Total	117	2.30	1.422	0.131	2.04	2.56
5	FGG	51	2.57	1.616	0.226	2.11	3.02
	PPG	66	1.88	1.365	0.168	1.54	2.21
	Total	117	2.18	1.512	0.140	1.90	2.46
6	FGG	51	2.61	1.710	0.239	2.13	3.09
	PPG	66	1.39	1.239	0.152	1.09	1.70
	Total	117	1.92	1.577	0.146	1.63	2.21
7	FGG	51	1.902	1.7579	0.2462	1.408	2.396
	PPG	66	1.121	1.1131	0.1370	0.848	1.395
	Total	117	1.462	1.4756	0.1364	1.191	1.732
8	FGG	51	1.294	1.4463	0.2025	0.887	1.701
	PPG	66	1.091	1.1297	0.1391	0.813	1.369
	Total	117	1.179	1.2755	0.1179	0.946	1.413
9	FGG	51	1.27	1.733	0.243	0.79	1.76
	PPG	66	0.94	1.162	0.143	0.65	1.23
	Total	117	1.09	1.442	0.133	0.82	1.35
10	FGG	51	0.41	1.043	0.146	0.12	0.71
	PPG	66	0.50	0.916	0.113	0.27	0.73
	Total	117	0.46	0.970	0.090	0.28	0.64
11	FGG	51	0.41	1.043	0.146	0.12	0.71
	PPG	66	0.18	0.493	0.061	0.06	0.30
	Total	117	0.28	0.786	0.073	0.14	0.43
12	FGG	51	0.12	0.475	0.067	-0.02	0.25
	PPG	66	0.18	0.493	0.061	0.06	0.30
	Total	117	0.15	0.485	0.045	0.07	0.24

FGG: free gingival graft, PPG: palatal pedicle graft

####  Clinical Measurements

 The surface area of the graft was calculated at baseline and three-month follow-up. The values were 171.05 ± 20.61 mm^2^ and 139.94 ± 21.02 mm^2^ in the PPG group and 225.41 ± 20.18 mm^2^ and 195.75 ± 25.49 mm^2^ in the FGG group, respectively. The groups did not show any significant differences in surface shrinkage changes either at baseline (*P* = 0.068) or three months after surgery (*P* = 0.103) (Table 4).

**Table 4:** Surface shrinkage at baseline and three months after surgery

**Surface shrinkage **		**N**	**Mean**	**Standard deviation**	**Standard error**	**95% Confidence interval for mean**		***P*** ** value**
						**Lower bound**	**Upper bound**	
Baseline	FGG	19	171.05842	89.876442	20.619070	127.73936	214.37748	0.068
	PPG	21	225.41710	92.503814	20.185987	183.30986	267.52433	
	Total	40	199.59673	94.192090	14.893077	169.47263	229.72082	
Three-months	FGG	19	139.94879	91.625930	21.020430	95.78650	184.11107	0.103
	PPG	21	195.75805	116.835198	25.495531	142.57530	248.94079	
	Total	40	169.24865	108.035338	17.081887	134.69727	203.80003	

FGG: free gingival graft, PPG: palatal pedicle graft.

 Compared with the FGG group, the PPG group exhibited significantly lower KTWs at baseline (*P* = 0.002), but the difference was not significant at one- and three-month follow-up evaluations (Table 5), indicating a higher increase in the width of keratinized mucosa in the PPG group.

**Table 5:** Keratinized tissue width at baseline and one- and three-month follow-ups

**Keratinized tissue width**		**N**	**Mean**	**Standard deviation**	**Standard error**	**95% Confidence interval for mean**		***P*** ** value**
						**Lower bound**	**Upper bound**	
Baseline	FGG	54	0.694	1.2148	0.1653	0.363	1.026	.002
	PPG	66	0.152	0.6383	0.0786	-0.005	0.308	
	Total	120	0.396	0.9764	0.0891	0.219	0.572	
One-month	FGG	54	4.94	1.664	0.227	4.49	5.40	.176
	PPG	66	5.33	1.461	0.180	4.97	5.69	
	Total	120	5.16	1.561	0.143	4.88	5.44	
Three-months	FGG	54	4.70	1.667	0.227	4.25	5.16	.783
	PPG	66	4.62	1.596	0.196	4.23	5.01	
	Total	120	4.66	1.622	0.148	4.37	4.95	

FGG: free gingival graft, PPG: palatal pedicle graft.

 The depth of the vestibule at the baseline (*P* = 0.006) and in the follow-ups of one (*P* < 0.001) and three months (*P* < 0.001) was significantly higher in the PPG group than in the FGG group (Table 6).

**Table 6:** Vestibular depth at baseline, one and three months follow-up

**Vestibular depth**		**N**	**Mean**	**Standard deviation**	**Standard error**	**95% Confidence interval for mean**		***P*** ** value**
						**Lower bound**	**Upper bound**	
Baseline	FGG	54	8.07	2.887	0.393	7.29	8.86	0.006
	PPG	66	9.77	3.645	0.449	8.88	10.67	
	Total	120	9.01	3.419	0.312	8.39	9.63	
One-month	FGG	54	8.02	2.375	0.323	7.37	8.67	< 0.001
	PPG	65	10.18	3.167	0.393	9.40	10.97	
	Total	119	9.20	3.024	0.277	8.65	9.75	
Three-months	FGG	54	8.00	2.802	0.381	7.24	8.76	< 0.001
	PPG	66	10.15	2.808	0.346	9.46	10.84	
	Total	120	9.18	2.993	0.273	8.64	9.72	

FGG: free gingival graft, PPG: palatal pedicle graft.

## Discussion

 A dental implant is usually covered by keratinized mucosa or mobile alveolar mucosa. It appears that the type of connective tissue beneath the epithelium determines its specificity (keratinized or non-keratinized). Therefore, the transplantation of connective tissue from the subepithelial palatal area to the peri-implant non-keratinized epithelium is at least partly responsible for keratinization induction.^15,16^ Recent evidence has shown that the durability of peri-implant tissues, and therefore the success of implant therapy, is determined by both the thickness of soft tissue and the peri-implant KTW.^17^ A lack of sufficient KTW surrounding dental implants has been linked to increased plaque accumulation, tissue inflammation, mucosal recession and/or attachment loss, patient discomfort, marginal bone loss, bleeding on probing, and lower patient esthetic satisfaction.^3,17,18^

 FGGs, connective tissue grafts, pedicle grafts, and apically positioned flaps have all been used to increase keratinized mucosa around implants.^19-21^ In addition to KTW formation, tissue shrinkage, and postoperative morbidity are also critical factors to consider when choosing the appropriate method for soft tissue augmentation. The graft shrinkage is a natural occurrence resulting from wound contraction and muscle repositioning, typically occurring within the initial month following surgery.^12^ Postoperative morbidities after tissue augmentation around dental implants can include pain, swelling, bleeding, and infection. These complications can be managed with proper postoperative care, such as antibiotic therapy, pain management, and careful oral hygiene.^22^ This randomized controlled clinical trial investigated the modification of the augmented soft tissue around the implant performed using either FGG or PPG and the patient-reported postoperative outcomes.

 We found no difference in the number of painkillers consumed by the patients in the study groups (except on the seventh day), but the PPG group patients reported significantly less pain from days 3 to 8 than the FGG group, when the NAS was analyzed. Less pain and morbidity can be attributed to the proximity of the donor and recipient sites in the PPG technique. Consistent with our findings, Elkhaweldi et al^10^ found PPG grafts less invasive with fewer morbidities than apically positioned flap, connective tissue grafts, and FGGs.

 In a study by Thoma et al,^23^ patients receiving FGGs reported the highest pain and discomfort in the first three days compared to other surgical techniques (apically positioned flap, subepithelial connective tissue graft, etc).

 According to a review study by Bassetti et al,^17^ shrinkage is expected to range from 0.20 to 3.06 mm,^24-26^ with rates up to 50.7%.^27^ According to another study, using the FGG technique resulted in a tissue width shrinkage within the mean range of 38%‒45%.^28^ However, the current study found 18% and 13% tissue shrinkage in FGG and PPG groups from baseline to three months of follow-up, respectively. Thoma et al,^29^ in a pilot study, observed a 16.8% shrinking rate of FGG grafts in the canine area of edentulous patients after three months. In their subsequent clinical trial, the shrinkage rate after three months was reported as 18.7%, consistent with our findings.Differences in surgical techniques and materials could explain the observed variability. For instance, it appears that combining APPTF (apically positioned partial thickness flap) with FGG, SCTG (subepithelial connective tissue graft), or XCM (xenogeneic graft material) results in less postoperative shrinkage than with other techniques like APPTF + AMDA (allogeneic graft materials). Another study reported that FGGs are associated with increased tissue shrinkage and a higher risk of necrosis. However, PPGs showed less tissue shrinkage because of the vascular connections remaining from the palatal area, graft thickness, and optimal quality.^30^ In addition, it is important to consider that the varying time points used as a baseline and the different follow-up periods may have impacted the outcomes. It is widely acknowledged that the shrinkage rate is significantly higher during the initial month following surgery.^31,32^ This trend persists at a lower magnitude for up to six months.^32^ Other factors that can contribute to surface shrinkage are the degree of muscle tension and the stability of the graft in the recipient area, as well as the graft thickness. Grafts with a thickness > 1.5 mm have a higher likelihood of primary shrinkage and necrosis risk. On the other hand, grafts with a thickness < 1.5 mm are more prone to secondary shrinkage. In this study, similar to Thoma and colleagues’, a 1.5-mm-thick graft was harvested from the palate and implanted into the recipient area.^29^

 The current study found that both treatment groups showed improvements in KTW, but the PPG method appeared more effective. According to Elkhaweldi et al,^10^ if at least 0.5 mm of keratinized tissue was present preoperatively, apical repositioning flaps could improve the thickness of keratinized tissue before implant implantation. Autogenous FGGs can be a viable alternative if the patient had less than 0.5 mm of keratinized tissue before the procedure. Bassetti et al^17^ reported an increase in the depth of the vestibule after soft tissue augmentation with FGG; however, it was not statistically significant. They found a relative, proportional association between the increase in KTW and higher vestibular depth.

 Due to the short follow-up period in this study, further evaluations should be conducted to compare the clinical outcomes of FFG and PPG methods over longer periods. Furthermore, using a split-mouth design for evaluations instead of paralleling can potentially mitigate confounding factors related to individual differences.

## Conclusion

 According to the present study, PPGs resulted in increased keratinized mucosal width and vestibular depth, with lower postoperative pain levels. However, the surface shrinkage and the number of painkillers consumed by the patients were comparable in both techniques.

## Acknowledgments

 This study was scientifically and technologically supported by the Tehran University of Medical Sciences.

## Competing Interests

 The authors declare no competing interests.

## Consent for Publication

 Not applicable.

## Data Availability Statement

 The data from the reported study are available upon request from the corresponding author.

## Ethical Approval

 The study protocol was approved by the Ethics Committee of Tehran University of Medical Sciences (ethical code: IR.TUMS. DENTISTRY.REC.1401.079).
